# Supplementary figures and images for: Tumor purity adjusted beta values improve biological interpretability of high-dimensional DNA methylation data
Source: PLoS One. 2022 Sep 9;17(9):e0265557. doi: 10.1371/journal.pone.0265557 (PMC9462735; doi:10.1371/journal.pone.0265557)

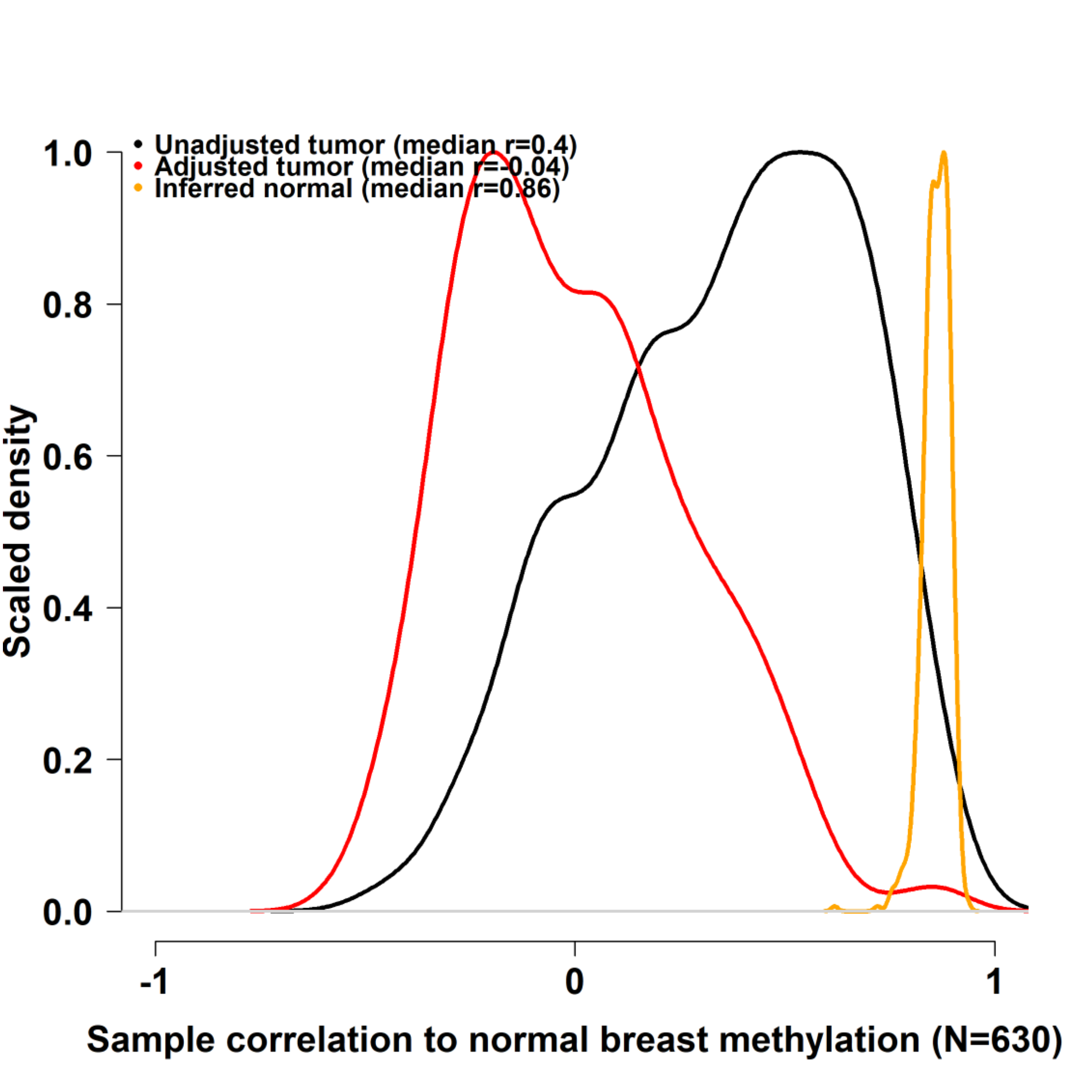

Supplement: S1 Fig — (TIFF) [file pone.0265557.s001.tiff]

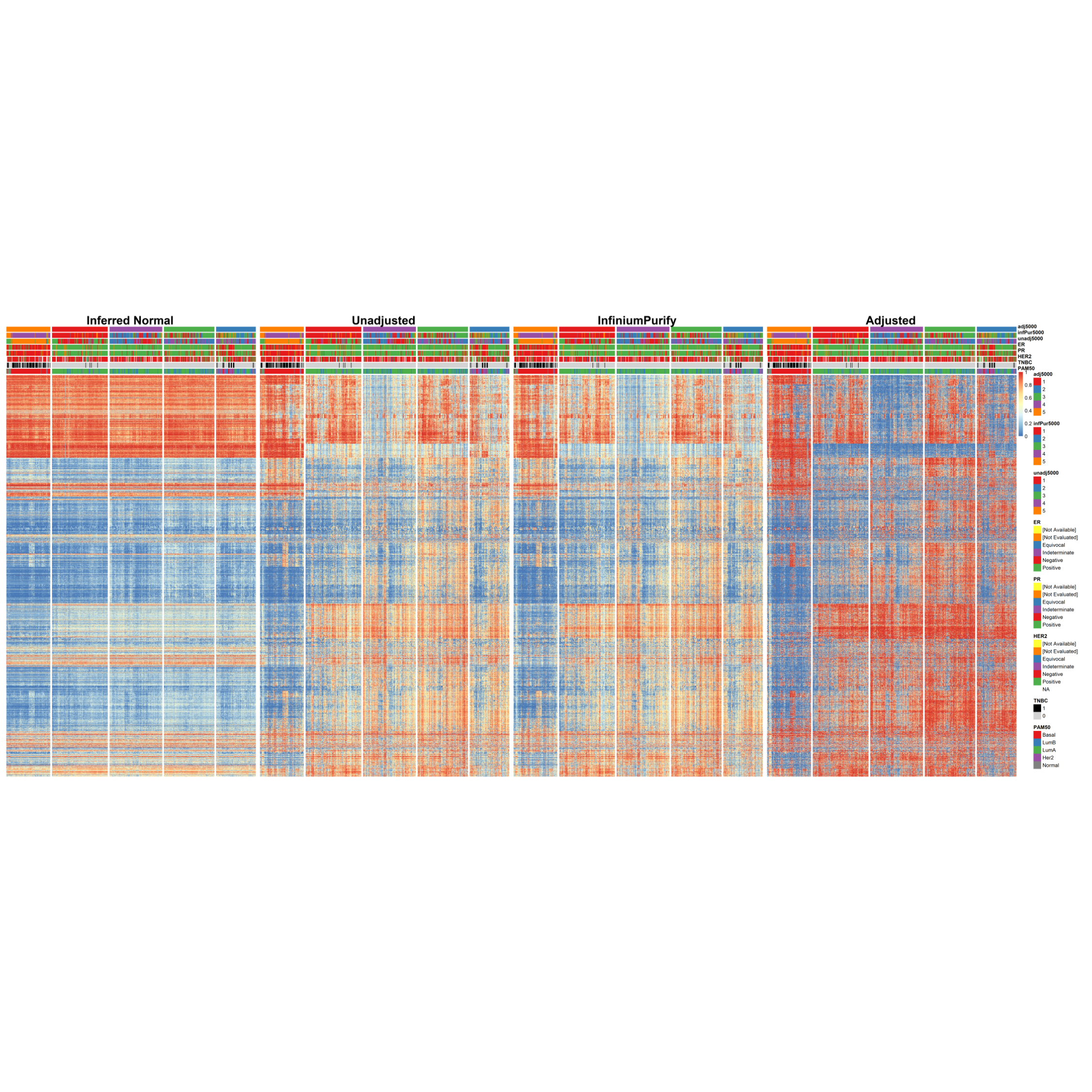

Supplement: S2 Fig — (TIFF) [file pone.0265557.s002.tiff]

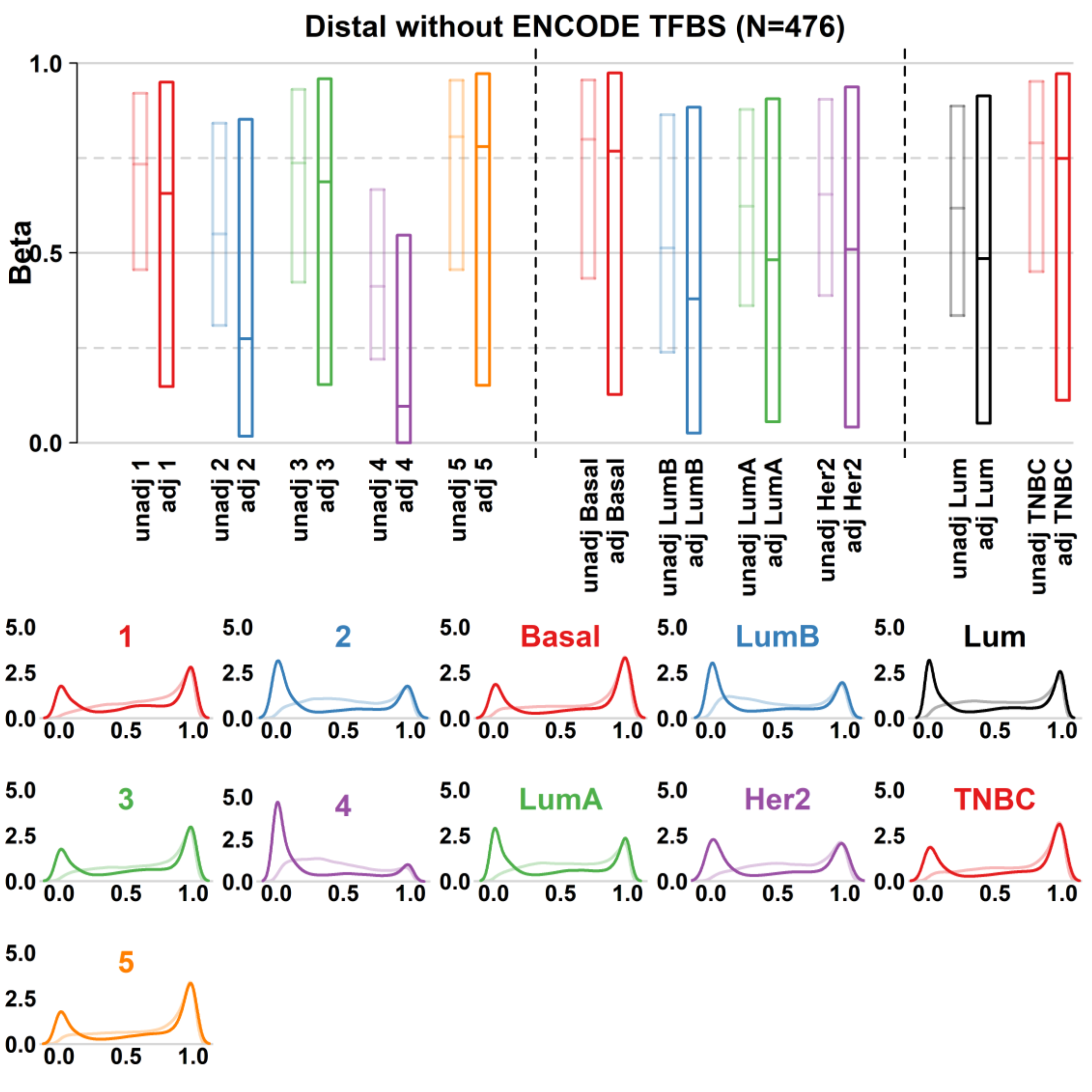

Supplement: S3 Fig — (TIFF) [file pone.0265557.s003.tiff]
